# Reaction to norm transgressions and Islamization threat in culturally tight and loose contexts: a cross-cultural comparison of Germany versus Russia

**DOI:** 10.1007/s40167-018-0073-3

**Published:** 2018-12-13

**Authors:** Liza Prentice, Johannes Klackl, Dmitrij Agroskin, Igor Grossmann, Yuri Alexandrov, Vladimir Apanovich, Boris Bezdenezhnykh, Eva Jonas

**Affiliations:** 1 Department of Psychology, Social Psychology, University of Salzburg, Hellbrunner Str. 34, 5020 Salzburg, Austria; 2 Department of Psychology, University of Waterloo, 200 University Avenue West, Waterloo, ON N2L 3G1 Canada; 3 Laboratory of Neural Bases of Mind, Institute of Psychology, Russian Academy of Sciences, Yaroslavskaya Str. 13, Moscow, Russia 129366; 4 Department of Psychology, National Research University Higher School of Economics, 20 Myasnitskaya Ulitsa, Moscow, Russia 101000

**Keywords:** Threat, BIS, BAS, Tightness, Need for tightness

## Abstract

Prior research shows that North Americans and Western Europeans react to threats with defensive strategies based on behavioral approach vs. inhibition systems (BAS/BIS)—i.e., a desire to approach a goal or to avoid a threat. In the present research, we explored whether this phenomenon is more pronounced in tight cultures (e.g., Germany) as compared to loose cultures (e.g., Russia), testing how Germans and Russians respond to societal threats. We expected that due to the higher levels of cultural tightness, Germans would show stronger defensive reactions to threats than Russians. Additionally, we investigated the role of need for tightness (i.e., need for strict regulation of social order) in threat management processes. In Study 1, Germans recalling violations of societal norms produced stronger rightward bias on the line bisection task than Russians, indicative of greater BAS activation in Germans than in Russians. In Study 2, we used frontal alpha asymmetry, providing the first cross-cultural test of BIS-BAS reactions utilizing neuronal markers. In this study, presentation of societal threat in a video portraying Islamic immigration as a large-scale violation of social norms led to higher BIS activation among Germans than among Russians, if their need for tightness was high. We discuss the role of tightness, need for tightness, and type of threat for cross-cultural particularities of threat-induced motivational shifts.

## Introduction

In the last decade, citizens in many European countries have faced a range of societal threats. For instance, after the conflicts in Ukraine over the course of the last years, Russian citizens were disappointed by several rounds of sanctions imposed by the EU, United States, and other countries. External pressure and increasing political tension accompanied by deterioration of the Russian economic situation have had serious repercussions on the prevailing climate in Russia (Ivanov et al. 2016). The years were also not easy for Germany. Recent events in Germany include the shooting in the shopping mall in Munich on July 22, 2016, and the Berlin market massacre on December 19, 2016. Combined with the massive influx of refugees from the Middle East, those incendiary tragedies had a strong negative impact on the citizens’ feeling of safety, exacerbated by mass media. Concerns about such threats can impact individuals on two different levels (Huddy et al. 2016). On the one hand, people see such situations as interpersonal threats to their individual well-being. On the other hand, these situations can constitute societal threats to a larger social group and undermine groups’ well-being. For example, for many Russian citizens, the international sanctions on Russia resulted in unemployment and financial difficulties (Khamatkhanova 2015). Furthermore, such sanctions continue to foster the growing isolation of Russia from Western countries and hinder its cooperation with the latter (Wang 2015).

In the present paper, we want to shed light on how culture influences person’s reactions to societal threats. In this context, cultural tightness—i.e., the strength of social norms (Gelfand et al. 2006), seems to be particularly relevant. Since tightness can emerge at a societal level as a consequence of past threat (Gelfand et al. 2011), it is likely to determine peoples’ cognition and behavior, as well as neural activity in the face of a current threat. The present research aims to compare people from Germany and Russia, cultures with different levels of tightness (Gelfand et al. 2011), in their threat responses.

### Culture and threat vigilance

Psychological research suggests that people react to societal and interpersonal threats using a variety of defensive behaviors and cognitions. For example, threat promotes ethnocentrism (e.g., Burke et al. 2010), reliance on stereotypes (Bodenhausen et al. 1994), and punitiveness against criminals (e.g., Proulx et al. 2012). People appear to use these kinds of defensive strategies with the aim to relieve anxiety caused by perceived threats and to restore equanimity (Jonas et al. 2014). Similar defensive intentions have been observed across a range of cultures (e.g., Heine et al. 2002; Tam et al. 2007), suggesting the universality of defensiveness in the face of threat.

At the same time, cultural particularities appear to influence specific threat management processes. Depending on cultural norms, political ideology, values, and religious beliefs, problematic situations can be perceived as more or less threatening and cause defensive reactions of different strength (e.g., Sallivan and Nonaka 1988; Yang et al. 2013; Schneider and De Meyer 1991). For example, cultural values such as solidarity and pride have been shown to facilitate effective coping with experienced threatening events (Moscardino et al. 2007). Similarly, cultural differences in independent versus interdependent self-construal modulate reactions to a self-related threat such that independent self-construal was related to stronger self-protective responses (Brockner and Chen 1996).

Given the cultural differences between Western countries and Russia (e.g., Kühnen et al. 2001; Naumov 1996), we can expect different reactions to threats also in these cultures. For example, prior research demonstrates that Russians display less distress while reflecting over a recent negative experience compared to Americans (Grossmann and Kross 2010). Further, it was shown that Russian culture promotes greater vigilance in response to negative stimuli (in terms of time spent and speed of stimuli recognition) as compared to Western cultures (Grossmann et al. 2012).

In the present paper, we argue that cultural tightness is a key factor underlying cultural differences in people’s responses to societal threats. As compared to looseness, tightness is defined as the strength of social norms and sanctions typical for the country (Gelfand et al. 2006), which is influenced by numerous distal ecological and historical threats, and socio-political practices (Gelfand et al. 2011). Tight cultures like Germany can generally be described as cultures with many strict social norms and low tolerance for deviant behavior, while loose cultures like Russia^1^ have few social rules and accept in general more deviant behavior (Gelfand et al. 2011). Tightness-looseness affects many areas of life: Tight countries have stricter parent–child-relationships (e.g., Halloway 1999), high-monitoring education strategies (e.g., Holzer 2000), and more media control (Sussman and Karlekar 2002). Moreover, development of several personality traits is at least partly affected by tightness-looseness: People in tight societies are generally more conforming, have a higher need for stability, and prefer to avoid risks (Gelfand et al. 2006).

Having such a big impact on individuals, tightness can also be a major factor in the context of threat responses. Prior research supports this idea, detecting culture-specific neuronal responses to a societal threat. Specifically, in a study investigating event-related potentials, culturally tight Chinese but not culturally loose US-Americans responded to social norm violation with an increased frontal N400—i.e., a neuronal marker of norm violation detection (Mu et al. 2015).

### Behavioral inhibition and approach systems

Research on the motivational dynamics underlying threat and defense suggests that responding to threat involves two main motivational systems: avoidance and approach (Jonas et al. 2014). For some individuals, threat activates a behavioral inhibition system (BIS; Gray 1982; Gray and McNaughton 2000). Accompanied by anxiety, BIS inhibits all current goal approach behavior and produces vigilance and avoidance, the adaptive purpose of which is to gather information about possible sources of threats and to avoid undesirable consequences (Jonas et al. 2014). At the neuronal level, BIS is associated with higher right frontal cortex activity (Agroskin et al. 2016; Harmon-Jones et al. 2010; Klackl et al. 2017).

For other individuals, threat activates behavioral approach system (BAS; Gray 1982; Gray and McNaughton 2000). BAS activation is associated with relative left frontal cortical activity (Harmon-Jones et al. 2010). Accompanied by either positive affects like hope, excitement, and eagerness (Corr et al. 2013) or negative affect like anger (Carver and Harmon-Jones 2009), BAS moves individuals towards a solution of the problem. However, if the solution is unavailable, people will undertake palliative or symbolic defensive strategies (e.g., affirming values), which do not solve the problem itself but nevertheless relieve anxiety (Jonas et al. 2014).

Whether people primarily engage BIS or BAS as a response to a threat seems to differ greatly depending on the type of threat at hand. For instance, freedom restrictions elicit immediate BAS activation (Steindl et al. 2016), whereas other threats like mortality salience and control deprivation cause immediate BIS activation (Agroskin et al. 2016; Klackl et al. 2017). Notably, the exact determinants of whether and when threat leads to BIS or BAS activation remain unclear, raising the question how contextual factors such as cultural tightness impact BIS/BAS activation.

### The role of cultural tightness for approach-avoidance motivations

There are theoretical reasons as well as empirical evidence for the impact of tightness on threat management. In general, we assume that people from tight cultures are more defensive in the face of threats than people from loose cultures. Depending on the nature of a threat, people from tight cultures may react to it either with higher BIS or higher BAS activation, as compared to people from loose cultures.

Tightness may amplify BIS-related responses to threats because people in tight societies have a clear idea of appropriate behavior and low tolerance of deviant behavior. They advocate established rules and expect others to follow them. Accordingly, people in tight cultures may experience situations as more threatening than people in less tight (i.e., loose) cultures because their views of “how things should be” get violated more easily. According to the anxiety-to-approach model (Jonas et al. 2014), it is possible that people from tight countries respond with higher BIS activation to a threat compared to people from less tight countries because threats invoke more substantial conflict and contradict expectations to a greater extent. Previous research hints to a possible connection between the level of tightness and BIS reaction to a threatening stimulus: Mrazek et al. (2013) relate tightness to a specific genetic polimorphism,^2^ which was previously associated with increased harm avoidance (Munafò et al. 2005), anxiety (Lonsdorf et al. 2009), and attentional bias to negative information (Munafò et al. 2009) when facing a stressful event. All these parameters correspond to the typical outputs of BIS.

Tightness can also amplify BAS-related responses to threats as it provides clear rules and norms that clarify how one should behave to overcome a threat and in this way reduces anxiety (Gelfand et al. 2006). In particular, it is possible that despite evaluating problematic situation as more threatening, people in tight (vs. loose) cultures can find ways to deal with problems more easily, which results in stronger approach motivation (i.e., BAS activation). Prior research provides some support for this hypothesis. For instance, individual difference studies have demonstrated that people with higher sensitivity to unfair treatment (a type of norm violation) experience more anger and outrage and demonstrate more prosocial behavior when facing a threat, which points to BAS-activation (e.g., Schmitt et al. 2009). Studies on Chinese samples demonstrate that people from this rather tight culture react to societal threats with high social coordination (i.e., the ability to effectively synchronize actions), which is another example of solution-oriented BAS responses (Mu et al. 2017).

Putting these insights together, we expect people from tight cultures to show stronger threat-specific defensive reactions, which can be aligned either with BIS or BAS activation.

### Tightness versus need for tightness

Besides tightness itself, one can also speculate that the strength of BIS-BAS reactions might be determined by the extent to which people report being in need for new norms and solutions, a construct that we call need for tightness. Whereas tightness typically describes the perception of existing norms and regulations in a society, need for tightness describes how strict norms and regulations *ought to be*. It is noteworthy that need for tightness does not have to correspond to the perceived level of societal tightness, similar to how perceived consensus in a culture does not need to correspond to personal beliefs (Zou et al. 2009). In fact, one may even expect compensatory processes where low perceived tightness in the society may correspond to high need for tightness. Interested in this new construct, we sought to explore whether need for tightness has an impact on reactions to societal threats. In particular, we speculate that people with high need for tightness may feel more anxiety and be more sensitive to societal threats because their need for regulation is violated. Thus, we assume that people with high need for tightness may show stronger BIS response in the face of threat, as compared to people with low need for tightness.

### The present research

The aim of the present research is to compare people with tight and loose cultural backgrounds in their reactions to societal threats, using German and Russian cultural contexts as a case study. We conducted two experiments to investigate culture-specific responses to societal threats. We predicted that people in culturally tight Germany would show a stronger defensive reaction to societal threats (i.e., BIS or BAS activation) than people in culturally loose Russia. We did not specify whether it would be BIS or BAS reaction because at the time of the data collection, there was no research demonstrating that societal threats caused one of these responses specifically. Additionally, we examined whether people with high need for tightness would show a stronger BIS reaction to societal threats than people with low need for tightness. Given the novelty of the need for tightness instrument, we treated this second part as exploratory.

In Study 1, participants were dealing with a threat directly related to existing rules: Violation of social norms. Participants recalled examples of norm violations. We explored subsequent BIS-BAS activity, using self-report measures of affect, and the line bisection task (Jewell and McCourt 2000), which is a perceptual measure of relative frontal activity (Nash et al. 2010). In Study 2, we used another type of societal threat and another measure of BIS-BAS activation. In this study, we induced threat, using a video clip suggesting that the influx of Muslim refugees would lead to global Islamization. Similarly to reactions to social norm violations, attitudes towards immigrants are related to the level of cultural tightness (e.g., Harrington and Gelfand 2014). However, the collective threat of global Islamization has a broader character and may require new norms for its regulation, which explains our interest in it. In Study 2, we investigated BIS-BAS reactions, using EEG to assess frontal brain activity as a neuronal marker of these processes. The line bisection bias and frontal EEG alpha asymmetry as markers of BIS-BAS activation were related in prior research (Nash et al. 2010). Thus, overall, we explored BIS and BAS, using both indirect and direct measures of neuronal activity associated with BIS-BAS processes.

## Study 1

In Study 1, we aimed to test if people from a tight culture (Germany) show stronger responses to a threat (i.e., BIS-BAS activity) compared to people from a less tight culture (Russia), using social transgressions as a threat. For exploratory reasons, we also investigated a possible impact of need for tightness. We conducted the norm violation manipulation twice, using two different perspectives: Participants had to imagine and describe, first, a situation when they broke a rule, and later on, a situation when they witnessed someone else break a rule.^3^

To investigate BIS-BAS activity, we used a behavioral measure of relative frontal hemisphericity: the line bisection task (Jewell and McCourt 2000). The LBT measures the extent to which people’s perception of horizontal lines is biased to the right or left visual fields. Right- or leftward bias reflects relatively higher activity of left and right frontal cortex, respectively. Thus, the LBT has previously been used as a measure of BIS and BAS states (Agroskin et al. 2016; Nash et al. 2010). Additionally, we measured self-reported BIS-related emotions.

### Method

#### Participants

Participants were 71 people from Russia and 67 people from Germany/Austria (53 Germans).^4^ Since German and Austrian cultures share a great overlap in their mentalities as well as a similar level of tightness (Gelfand et al. 2011) we combined participants from these cultures into one group. The age of participants ranged from 17 to 62 in Russia (*M *= 25.20, *SD* = 10.72) and from 17 to 65 in Germany/Austria (*M *= 26.49, *SD* = 9.31). In both cultural groups, there were more female than male participants: 64 and 50, respectively. The majority of participants were students (*N*_RU_ = 36; *N*_GER/AU_ = 50). The study was approved by the ethics committee of the University of Salzburg. All participants signed informed consent and could withdraw participation at any point, although no participant made use of this option.

#### Procedure and materials

The Russian- and German-language questionnaires were presented via Questback software (QuestBack, Oslo, Norway). All measures were translated from German to Russian and back by independent bilingual graduate students, following established procedures (Grossmann and Na 2014). We recruited participants in the year 2013 through social networks Facebook and Vkontakte (a Russian on-line social network system comparable to Facebook in design and features). Participation in the study was voluntary and anonymous. All participants were informed about the confidentiality of their data.

##### Line bisection task (LBT)

The questionnaire included four parts. After a brief introduction and instructions, participants completed the first LBT (*α*_RU_ = .98, *α*_GER/AU_ = .76), which we used as a baseline measure. In this task, participants had to mark the perceived center point of ten horizontal lines, which varied in size and screen positions. The lines were between 45 and 100 mm long. Participants could bisect the lines at positions 1–100 (see Glicksohn and Kinberg 2009 who used a similar procedure). We calculated bisection bias by averaging deviations from the line centers (see Klackl et al. 2017). Negative scores indicated stronger leftward bisection bias or relative right hemisphericity indicative of avoidance motivation. Positive LBT-scores indexed higher BAS activation.

##### Manipulation and affect measure

Following the first LBT, participants read a definition of social norms accompanied with several examples. Then, we asked them to recall and describe a past situation in which they broke an existing social rule (Norm violation: Transgressor) and to answer two open-ended questions about that situation (“Please try to remember a situation in which you violated a certain social norm. Describe this situation in a few sentences. What were your thoughts and feelings in this situation? How did other people react?”). Right after that, participants completed the second LBT (*α*_RU_ = .99, *α*_GER/AU_ = .75), which was identical to the first LBT except that the lines were shuffled. Next, participants assessed how much each of 18 given affect items reflected their feelings in the described situation, using a 5-point Likert scale (from 1, “absolutely not” to 5, “absolutely”, see “Appendix 1”). We were interested in four items that represented an affective side of BIS reaction and corresponded to the BIS Affect Scale (Agroskin et al. 2016), namely uncertainty, anxiety, nervousness, and confusion (*α*_RU_ = .79, *α*_GER/AU_ = .80). Then, participants evaluated the perceived severity of the norm violation in the described situation (one item “How severe was the norm violation you described” from 1, “absolutely not severe”, to 6, “very severe”).

In the third part of the questionnaire, participants had to recall a situation in which they witnessed someone else breaking a social rule (Norm violation: Observer). After giving a brief description, participants answered two open-ended questions: “What were your thoughts and feelings in this situation?” and “How did you react to this situation?” Following that, participants completed the third LBT (*α*_RU_ = .96, *α*_GER/AU_ = .69), the affect scale (*α*_RU_ = .74, *α*_GER/AU_ = .79 for the BIS subscale), and evaluated the severity of the norm violation. We balanced the order of “Norm violation: Transgressor” and “Norm violation: Observer” parts between the subjects.

##### Tightness and need for tightness

The fourth part contained demographics, several traits measures,^5^ the Edinburgh handedness inventory, *α*_RU_ = .53, *α*_GER/AU_ = .87 (Oldfield 1971), and a short compliance check. Then, we measured a perceived level of cultural tightness, using the Tightness-Looseness Scale (Gelfand et al. 2011). Example items were “There are many social norms that people are supposed to abide by in [the country]” or “People in [the country] almost always comply with social norms.” Responses were given on a 1 (“Totally disagree”) to 6 (“Totally agree”) scale. The original six-item version of the scale exhibited a low level of internal consistency (*α*_RU_ = .45, *α*_GER/AU_ = .49). We removed the fourth item “People in this country have a great deal of freedom in deciding how they want to behave in most situations” (reverse coded) in order to improve the internal reliability (*α*_RU_ = .55, *α*_GER/AU_ = .60).^6^ Additionally, we measured need for tightness, using a four-item scale inspired by the Tightness-Looseness scale. The wording of the items was changed to indicate how things ought to be in the respective country (e.g., “There should be many social norms that people are supposed to abide by in [the country]”). The modified version of the scale (*α*_RU_ = .80, *α*_GER/AU_ = .68) is presented in “Appendix 2”.

### Results and discussion

As a first step, we compared the two cultural samples regarding the level of tightness and need for tightness. Consistent with previous research (Gelfand et al. 2011), German/Austrian participants perceived their culture to be tighter than Russian participants did (*M*_GER/AU_ = 4.33, *SD*_GER/AU_ = 0.57, *M*_RU_ = 3.50, *SD*_RU_ = 0.67), *t*(133) = 7.83, *p *< .001, *d* = 1.33.^7^ Conversely, Germans/Austrians expressed lower need for tightness than Russians (*M*_GER/AU_ = 3.51, *SD*_GER/AU_ = 0.80, *M*_RU_ = 3.93, *SD*_RU_ = 0.91), *t*(120) = − 2.69, *p *= .008, *d* = 0.49. Notably, the two constructs appeared orthogonal, *r*_RU_ = .23, *p*_RU_ = .08, *r*_GER/AU_ = .18, *p*_GER/AU_ = .17.

We also compared how participants with different cultural backgrounds evaluated the severity of different types of norm violation (transgressor vs. observer). Participants from both cultural groups rated situations in which somebody else broke a rule as significantly more severe than situations in which they broke a rule themselves (*M*_GER/AUtrans_ = 3.12, *SD*_GER/AUtrans_ = 1.40, *M*_GER/AUobs_ = 3.91, *SD*_GER/AUobs_ = 1.36, *M*_RUtrans_ = 2.90, *SD*_RUtrans_ = 1.51, *M*_RUobs_ = 4.06, *SD*_RUobs_ = 1.48), *F*(1, 136) = 57.20, *p * < .001, *η*_p_^2^ = .30. Importantly, the main effect of culture (*p *= .863), as well as the Culture × Norm violation type interaction (*p *= .160), was non-significant. Inclusion of the order of the manipulations as a covariate in the analysis had no significant influence on the results.

**Fig. 1:**
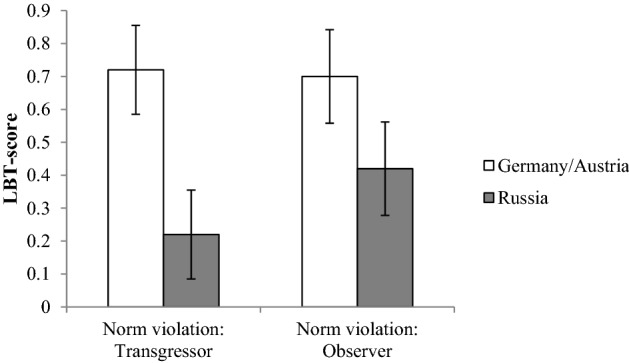
After recalling social transgressions, Germans/Austrians showed higher LBT-scores (i.e. more BAS-related approach) compared to Russians. Error bars represent ± 1 SEM

Next, we tested whether people from Germany and Austria differed in their line bisection responses to threat from people from Russia. We employed a 2 (Russia vs. Germany/Austria) × 2 (LBT 2 vs. LBT 3) mixed effects general linear model with the first LBT as a covariate. We included only right-handed participants in the analysis to control for hemispheric differences correlated with handedness (e.g., Drake and Myers 2006). To correct for outliers, we took only the LBT-trials in which participants put the mark at the central 20 percent of the lines. The resulted sample consisted of 57 Russians and 57 Germans/Austrians. The analysis revealed a significant main effect of culture, *F*(1, 111) = 4.85, *p *= .030, *η*_p_^2^ = .042, showing that German/Austrian participants responded with higher LBT-scores indicative of increased approach (BAS) compared to Russian participants after both norm violation types (*M*_GER/AUtrans_ = 0.72, *SD*_GER/AUtrans_ = 1.15, *M*_RUtrans_ = 0.22; *SD*_RUtrans_ = 1.14, *M*_GER/AUobs_ = 0.70, *SD*_GER/AUobs_ = 1.26, *M*_RUobs_ = 0.41, *SD*_RUobs_ = 1.40), see Fig. 1. The effect of the norm violation type (*p *= .828), as well as the Culture × Norm Violation Type interaction (*p *= .220), was non-significant. We conducted a similar analysis, controlling for the order of the manipulations. Inclusion of the covariate had no impact on the reported results.

To test if tightness could explain the revealed effect of culture on bisection bias, we employed two mediation analyses, using the PROCESS macro for SPSS (Model 4; Hayes 2016). The analyses explored the underlying mechanism by which a predictor (culture) influenced an outcome (LBT 2 or LBT 3) through a mediator (cultural tightness). Contrary to our expectations, the analyses did not reveal an indirect effect of culture through tightness neither on LBT 2, *b* = − 0.16; SE = 0.15; 95% BC CI [− 0.50; 0.10], nor on LBT 3, *b* = − 0.14; SE = 0.31; 95% BC CI [− 0.99; 0.29].

For exploratory reasons, we tested whether need for tightness might moderate the impact of culture on the line bisection responses and performed a moderation analysis, using the PROCESS macro for SPSS (Model 1; Hayes 2016). We considered culture as a predictor, need for tightness as a moderator, and LBT-scores as an outcome. We performed separate analyses for the transgressor and the observer condition. However, we found no Culture × Need for Tightness interactions at LBT 2 (*p* = .712) or LBT 3 (*p* = .385).

As another marker of BIS activation, we used self-reported BIS affect. We conducted a similar 2 (Russia vs. Germany/Austria) × 2 (transgressor vs. observer) mixed effects general linear model. We included right-handed and left-handed participants in the analysis. The test revealed a main effect of norm violation type, *F*(1, 136) = 7.33, *p *= .008, *η*_p_^2^ = .051, indicating that recalling situations in which one broke a rule caused more BIS-related emotions than situations when one observed how somebody else broke a rule. However, this effect appeared to be non-significant when controlling for the order of the manipulations, *F*(1, 135) = 0.91, *p *= .342, *η*_p_^2^ = .007. Utilizing both analytical strategies, we found neither a main effect of culture nor a Culture × Norm Violation Type interaction, all *p*s > .275 Mean values demonstrated that participants from both cultural groups did not express strong BIS-related emotions (*M*_GER/AUtrans_ = 2.28, *SD*_GER/AUtrans_ = 0.99, *M*_RUtrans_ = 2.32, *SD*_RUtrans_ = 1.03, *M*_GER/AUobs_ = 2.01, *SD*_GER/AUobs_ = 0.91, *M*_RUobs_ = 2.09, *SD*_RUobss_ = 0.97).

#### Additional analyses

We also examined if the two cultural groups differed in any other emotions measured by the affect scale. We observed a significant main effect of the culture regarding sadness, *F*(1, 132) = 22.94, *p *< .001, *η*_p_^2^ = .15, indicating that Russian participants experienced more sadness compared to German participants independent of the transgression type (*M*_GER/AUtrans_ = 1.67, *SD*_GER/AUtrans_ = 1.06, *M*_RUtrans_ = 2.37, *SD*_RUtrans_ = 1.40, *M*_GER/AUobs_ = 1.81, *SD*_GER/AUobs_ = 1.08, *M*_RUobs_ = 2.84, *SD*_RUobs_ = 1.48). The transgression type main effect was also significant, *F*(1, 132) = 5.74, *p *= .018, *η*_p_^2^ = .042, showing that participants in the “Norm violation: Observer” condition expressed more sadness. However, this effect appeared to be non-significant when controlling for the order of the manipulations, *p *= .119. We did not find any significant results with regard to the Culture × Norm Violation Type interaction, all *p*s > .175

#### Discussion

In line with our hypothesis, German/Austrian participants showed a stronger threat response than Russian participants. Specifically, Germans/Austrians exhibited stronger rightward bisection bias indicative of BAS activation in response to norm violations, as compared to Russians. BAS rather than BIS reaction to such societal threat as norm violation is in line with prior research. For instance, previous studies demonstrated that anger and outrage, which are emotions associated with BAS, are typical reactions to norm violation (e.g., Boll 1998). However, individual differences in tightness did not mediate the effect of culture on BAS response. There are two issues to consider. First, the Tightness-Looseness Scale (Gelfand et al. 2011) we used in Study 1 measures individual differences, which may not necessarily correspond to cultural differences (Na et al. 2010). Second, the scale exhibited a rather low level of internal reliability, raising questions about measurement equivalence of this scale across cultures. We also did not find any effects of need for tightness.

Study 1 also showed that participants from both cultures assessed own transgressions as less severe than transgressions of others. This effect seems to be a simple example of cognitive dissonance reduction (Festinger 1957). Additionally, the study revealed that Russians experienced more sadness than Germans in both conditions. Although our hypotheses did not touch upon sadness, we found this effect interesting also in the context of the present research because it sheds light on possible reactions to threats in Russia. Prior research demonstrates that Russians report more sadness experiences compared to Americans and hints to a special meaning of sadness in Russia (Chentsova-Dutton 2011; Consedine and Magai 2002). There is also some empirical evidence that Russians tend to brood and be melancholic (e.g., Grossmann and Kross 2010). Thus, higher sadness might reflect melancholic states typical for Russians.

The findings of Study 1 provide initial support to the prediction that reactions to threat should be more pronounced among tight cultures (Germany/Austria) relative to loose cultures (Russia). In Study 2, we wanted to replicate these results, using a direct measure of BIS-BAS activity and a broader societal threat.

## Study 2

Based on Study 1, Study 2 further compared Russians and Germans in their reaction to a societal threat and explored the possible effects of tightness and need for tightness. In contrast to Study 1, in which we used a small-scale manipulation of threat (i.e., self- and other-caused transgressions), we used a large-scale threat that should be perceived as a collective risk. For this reason, we used a video stimulus that created an impression of a growing Islamization of Europe. Whereas in Study 1 we relied on the line bisection task (LBT) as a marker of BIS-BAS responses, in Study 2, we used electroencephalography (EEG) to measure frontal alpha asymmetry, a neuronal index of left- and right-lateralization indicative of BIS and BAS processes. As in Study 1, we expected people from culturally tight Germany to respond more strongly to threat.

### Method

#### Participants

The sample consisted of 41 persons from Russia (21 males, 20 females) and 52 persons from Germany (10 males, 42 females) ranging in age from 17 and 54 in Russia (*M *= 22.54, *SD* = 7.75) and from 18 to 33 in Germany (*M *= 22.17, *SD* = 3.14). In both groups, the majority of participants were students (*N*_RU_ = 31; *N*_GER_ = 51). The study was approved by the ethics committee of the University of Salzburg. All participants signed informed consent and could withdraw participation at any point, although no participant made use of this option.

#### Procedure and materials

The data were collected in the year 2014 at the University of Salzburg and the Institute of Psychology of the Russian Academy of Sciences. We invited participants to the lab where they underwent EEG recordings and filled in an online questionnaire. All participants gave written informed consent to participate in the study and received monetary reward or course credits for participation.

##### Manipulation

After a brief introduction, participants underwent a 90-second EEG recording trial (the EEG procedure is described below in full detail). Then, participants watched threat and control videos in counterbalanced order. The threat video was about growing Islamization of Europe (Jack 2015). Accompanied by the song of a Muezzin, it painted a picture of a steep growth in Muslim population in Europe in the nearest future. According to the video, in 2050, Germany would be an Islamic country, and every fifth person in Russia would be a Muslim. The control video was about environment-friendly production and recycling of paper (Antalis 2015). The videos were presented in English with German or Russian subtitles. Both videos were about three minutes in length (2:39 and 3:03, respectively).

##### EEG recording

After each video, participants underwent a 90-second EEG recording. Participants were instructed to think about the video during the recording. Next, participants performed a passive 3-stimulus auditory oddball task for about two minutes. This task was used to assess whether culture and/or perceived tightness affected the oddball P300. No reliable effects were discovered. After this task, participants underwent another 90-second EEG recording. Thus, the interval between the two videos was about five minutes. To investigate possible carryover effects, we included the order of the videos as a covariate in our statistical analyses.

At the both study locations, we used the same14-channel Emotiv EEG neuroheadset and Emotiv TestBench software (Emotiv Systems Inc., San Francisco, CA, USA) to record data with a sampling rate of 128 Hz. We recorded data from sites AF3, AF4, F3, F4, F7, F8, FC5, FC6, P7, P8, T7, T8, O1, and O2. EEG data were re-referenced to an average of electrodes T7 and T8 and filtered (high/pass cutoff: 0.1 Hz, Slope: 24 db/Oct; low-pass cutoff: 30 Hz, Slope: 24 db/Oct). We calculated frontal asymmetry scores for each 90 s recording period by segmenting each 90 s segment into 2 s segments with 1.5 s overlap. We only included epochs in which the difference between two values in a moving 200 ms interval did not exceed 100 microvolts and the signal was between -100 and +100 microvolts. The 2 s segments were Fourier transformed (Fast Fourier Transform, 10% Hamming Window, frequency resolution 0.5 Hz), and the resulting power spectra were averaged. Finally, individual frontal alpha (8-13 Hz) asymmetry for each epoch was calculated (log F8 − log F7). These electrodes are known to provide the best fit in predicting behavioral responses on the line bisection bias (Nash et al. 2010).

To prepare alpha asymmetry data to the primary analyses, we calculated unstandardized residuals using pre-video asymmetry scores as predictors of post-video asymmetry scores. Negative values indexed relative right frontal asymmetry (RFA) and thus, indicated BIS. Positive values indexed relative left frontal asymmetry (LFA) and thus, were markers of BAS (e.g., Harmon-Jones et al. 2010).

##### Tightness and need for tightness

After the EEG-recording, participants completed an online questionnaire, which we created, using Questback software (QuestBack, Oslo, Norway). As in Study 1, we used the Edinburgh handedness inventory, α_RU_ = .84; α_GER_ = .72 (Oldfield 1971), the Tightness-Looseness Scale (α_RU_ = .63, α_GER_ = .55) without item 4 (Gelfand et al. 2011), and the Need for Tightness Scale (α_RU_ = .73, α_GER_ = .56). Next, participants completed several traits measures^8^ and described their thoughts about the videos. Finally, participants completed short manipulation and compliance checks. The seven-item manipulation check measured how alarming participants found the threat (α_RU_ = .86, α_GER_ = .78) and control (α_RU_ = .74, α_GER_ = .81) videos (e.g., “To what extent did the video make you feel worried?”) on a 1 (“Absolutely not”) to 5 (“Very”) scale. All measures were translated from German to Russian and back by independent bilingual graduate students, following established procedures (Grossmann and Na 2014).

### Results and discussion

Similar to Study 1, we first compared two cultural groups in the level of perceived tightness and need for tightness. In line with our expectations, Russians rated their culture as less tight than Germans did (*M*_RU_ = 3.72, *SD*_RU_ = 0.78, *M*_GER_ = 4.5, *SD*_GER_ = 0.47), *t*(91) = 5.92, *p *< .001, *d* = 1.21. At the same time, Russians showed higher need for tightness scores than Germans (*M*_RU_ = 3.81, *SD*_RU_ = 0.94, *M*_GER_ = 3.38, *SD*_GER_ = 0.69), *t*(91) = 2.55, *p *= .013, *d* = 0.52. As in Study 1, tightness and need for tightness were not correlated, *r*_RU_ = .17, *p*_RU_ = .30, *r*_GER/AU_ = .21, *p*_GER/AU_ = .13.

Prior to the main analysis, we also tested whether participants viewed Islamization as a threatening issue. The manipulation check revealed that participants in both cultures found the Islamization video more threatening than the paper video, as revealed by a significant main effect of threat (*M*_isl_ = 2.78, *SD*_isl_ = 0.70, *M*_pap_ = 1.80, *SD*_pap_ = 0.51), *F*(1, 90) = 11.61, *p *= .001, *η*_p_^2^ = .11, in the absence of a culture main effect or an interaction, all *p*s > .275.

To test our hypothesis about differences between loose Russia and tight Germany in reactions to threat, we employed a 2 (Russia vs. Germany) × 2 (threat vs. control video) mixed effects general linear model. After artifact rejection and exclusion of left-handed participants, the sample consisted of 24 Russians and 34 Germans. The analysis did not reveal any differences in alpha asymmetry between the Russian and the German sample, *F*(1, 56) = 1.51, *p *= .225, *η*_p_^2^ = .026, or between the two conditions, *F*(1, 56) = 0.19, *p *= .891, *η*_p_^2^ = .001. The Culture × Condition interaction was also not significant, *F*(1, 56) = 0.08, *p *= .931, *η*_p_^2^ = .001. Inclusion of the order of manipulations as a covariate did not change the results.

**Fig. 2:**
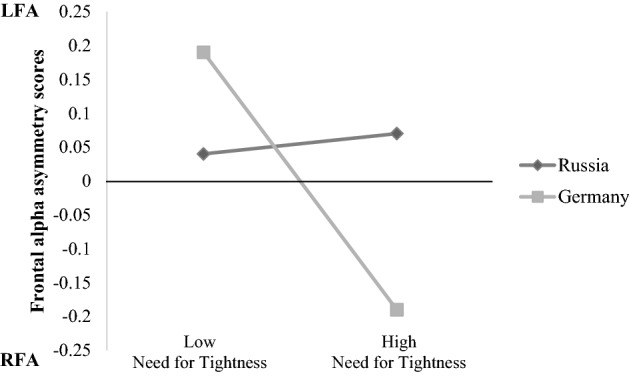
After watching a threatening video about Islamization, Germans showed lower alpha asymmetry scores (i.e. more BIS) compared to Russians, but only when need for tightness was high. *LFA* left frontal asymmetry, *RFA* right frontal asymmetry

For exploratory reasons, we tested whether need for tightness might determine the impact of culture on frontal alpha asymmetry and performed a moderation analysis, using the PROCESS macro for SPSS (Model 1; Hayes 2016). We considered culture as a predictor, need for tightness as a moderator, and frontal asymmetry scores as an outcome. We performed separate analyses for the experimental and the control condition. In the threat condition, we found a significant Culture × Need for Tightness interaction, see Table 1. There was a difference in alpha asymmetry scores between the two cultures, if need for tightness was high, *b* = − 0.29, SE = 0.11, *t*(54) = − 2.64, *p* = .010, but not low, *b* = 0.05, SE = 0.10, *t*(54) = 0.54, *p* = .59. In particular, German participants with high need for tightness had lower LFA scores indicating a stronger BIS reaction to the Islamization video than Russian participants. In Russia, need for tightness had no impact on frontal asymmetry scores, see Fig. 2.

**Table 1:** Frontal alpha asymmetry scores predicted from culture and need for tightness

Predictor	*b*	SE	*t*	*p*
Constant	− 0.03	0.04	− 0.80	.43
Need for tightness	− 0.12	0.05	− 2.53	.015
Culture	− 0.12	0.07	− 1.61	.11
Need for tightness × culture	− 0.21	0.09	− 2.40	.020

In the control condition, the moderation analysis revealed no significant effect (*p* = .709). All results remained the same when controlling for the order of manipulations.

As in Study 1, we compared the two cultural samples in experienced sadness for exploratory reasons. To do so, we used one item included in the manipulation check: “To what extent did the video make you feel sad?” We analyzed the data of all participants and employed a similar 2 × 2 mixed effects general linear model. We found a significant Culture × Condition interaction, *F*(1, 91) = 11.46, *p *= .001, *η*_p_^2^ = .11, showing that in the Islamization condition, Russian participants experienced more sadness than German participants, *p *< .001 (*M*_RU_ = 2.93, *SD*_RU_ = 1.23, *M*_GER/AU_ = 1.81, *SD*_GER/AU_ = 0.89). In the paper condition, there were no differences between Russian and German participants, *p *= .178.

#### Additional analysis of the open-ended questions

In Study 2, we also asked participants to describe their thoughts and feelings about the problem of growing Islamization of Europe. We assumed that this information might help us to understand the revealed differences in reactions to the threat. To analyze the answers, we recruited 34 Russian coders (30 females and 4 males; *M*_age_ = 23.81, *SD*_age_ = 3.86) and 26 German/Austrian coders (19 females and 7 males; *M*_age_ = 22.96, *SD*_age_ = 3.88). All statements were translated in Russian and German languages by bilingual graduated student, and then back-translated by a professional translator. Coders assessed the answers provided by Russian and German participants (*N*_RU_ = 41, *N*_GER_ = 52) without knowing the origin of the statements. Coders evaluated how abstract/concrete (from 1 “Absolutely abstract” to 5 “Absolutely concrete”), self-relevant (from 1 “Not self-relevant” to 3 “Very self-relevant”), and emotional (from 1 “Not emotional” to 5 “Very emotional”) the statements were. Coders were instructed that the statements ought to be considered as abstract if they described a general idea but no details that would specify that idea. The statements ought to be considered as self-relevant if they were related to a person herself—i.e., were relevant to her and touched on her own life. Before evaluating the statements, the coders read a detailed description of the three criteria (abstractness, self-relevance, and emotionality) and went through a training trial.

We employed three separate 2 (Origin of the coders: Russian vs. German/Austrian) × 2 (Origin of the statements: Russian vs. German) mixed effects ANOVAs. Coders from both cultures assessed Russian statements as significantly more emotional (*M*_RU_ = 3.25, *SD*_RU_ = 0.54, *M*_GER_ = 3.13, *SD*_GER/AU_ = 0.49), *F*(1, 58) = 10.22, *p *= .002, *η*_p_^2^ = .15, and less self-relevant than German statements (*M*_RU_ = 2.13, *SD*_RU_ = 0.24, *M*_GER_ = 2.18, *SD*_GER/AU_ = 0.26), *F*(1, 58) = 4.91, *p *= .031, *η*_p_^2^ = .08. Neither the coder origin main effect (*p*_em_ = .768, *p*_sr_ = .641), nor the interaction (*p*_em_ = .315, *p*_sr_ = .244) was significant. No significant effects were found regarding the statements’ abstractness.

#### Discussion

Study 2 revealed that compared to Russians, Germans reacted to Islamization threat with decreased left frontal alpha asymmetry indicative of BIS activation, but only if their need for tightness was high. Therefore, Study 2 showed that Germans reacted more strongly to threat, but this effect depended on need for tightness. Interestingly, need for tightness did not seem to influence defensive reactions in Russia. Thus, Study 2 provides preliminary evidence that individuals’ need for tightness might have an impact on threat-related responses and determine stronger BIS reactions in certain cultures and to certain threats, namely to a broad societal threat that cannot be regulated by existing social norms.

Additionally, Study 2 revealed noteworthy intercultural differences in how participants from Russia and Germany reasoned about growing Islamization. Russians wrote about the topic in a more emotional way but considered it less self-relevant than Germans. These findings correspond to previous research showing that Russians tend to reflect on their negative feelings and to keep more self-distance than Westerners do (Grossmann and Kross 2010). Further, as in Study 1, we demonstrated that Russians reacted to threat with higher self-reported sadness than Germans.

## General discussion

In the present paper, we address the question how people with Russian and German cultural backgrounds deal with societal threats they face in their daily lives. Specifically, we wanted to test whether defensive responses to threats based the activation of BIS and BAS would be more pronounced in culturally tight Germany, as compared to culturally loose Russia. Additionally, we explored a possible impact of need for tightness on responses to threats.

Two studies provide some evidence that Germans show stronger BIS-BAS as reactions to threats than Russians. In Study 1, people from Germany reacted to the violation of social norms with a stronger BAS activation than people from Russia. As discussed above, this pattern of results might be due to the well-developed social regulation typical for tight cultures, which helps people to approach and resolve problems. These findings demonstrate that stronger motivational reactions to norm violations might be another characteristic of tight societies.

In Study 2, we focused on reactions to a broader societal threat (growing Islamization of Europe). In this context, we did not observe significant differences between Russia and Germany directly. However, our exploratory analysis revealed that cultural differences were moderated by need for tightness: Compared to Russians, Germans displayed stronger BIS activation to Islamization threat, if their need for tightness was high.

Together, our results suggest that tightness plays a direct role for motivated reactions to small-scale norm violations (Study 1) but not for a large-scale, collective threats such as Islamization (Study 2). Speculating about some of the reasons for this divergence, we emphasize that Islamization constitutes a broader and more abstract threat than specific norm violation incidents. Although attitudes towards immigration itself are connected to tightness (Harrington and Gelfand 2014), growing Islamization as a jeopardy to cultural norms and values can represent an existential threat that cannot be regulated by existing norms. In other words, tightness may not be a relevant domain for an existential societal threat we tapped into in Study 2.

Interestingly, need for tightness selectively predicted right-frontal asymmetry indicative of BIS activation in the context of Islamization (Study 2) in Germans. This might be because in cultures where rules are usually present (i.e., Germany), the absence of clear rules to rely on may be particularly aversive, especially among individuals who desire them (i.e., have a high need for tightness).

To our knowledge, this is the first research that introduces the concept of need for tightness and investigates its role in threat regulation processes. Importantly, the weak correlation between need for tightness and perceived tightness indicates that need for tightness is an independent factor, which is important to consider in cross-cultural comparison. We demonstrate that need for tightness varies in different countries. Interestingly, Russian participants scored lower on tightness but higher on need for tightness, as compared to German participants. Moreover, our results suggest that need for tightness is a moderating factor in threat contexts that are devoid of clear norms and rules.

Our studies revealed that on average, Russians have a higher need for tightness than Germans. Assuming that high need for tightness in the face of Islamization would lead to greater BIS activation, we could expect Russians but not Germans to be in BIS state while dealing with Islamization issue. However, we could not show such reaction within the Russian sample. Although there are no social norms that could downregulate Islamization threat either in Russia or in Germany, the perception of that issue might be very different in the two cultures. Islam in Russia has a long history, and at least since the Tatar-Mongolian invasion in the Middle Ages, parts of the Islamic culture has become part of Russian mainstream culture (Malashenko and Nuritova 2009). For that reason, Russians might perceive it not as an acute threat requiring immediate social changes like in Germany, but as a continuous social phenomenon. Moreover, Russians live in a society with a relatively recent history of dramatic political and economic shifts and have higher anticipation of changes (Grossmann and Varnum 2011). For that reason, in Russia, societal threats might be perceived as less critical compared to the individual threats.

The two studies provided in this paper indicate that culturally tight (Germany) and loose (Russia) societies indeed respond differently to some threatening stimuli. One limitation is that these studies have relatively small samples, and the statistical tests conducted may suffer from low statistical power. The present studies were conducted before statistical power became highly salient in psychological science (Aarts et al. 2015). A high-power replication of the revealed effects should be one of the next steps in further investigations. Another step may involve an exploration which types of threat cause stronger defensiveness in tight societies. We focused on societal threats here because the concept of tightness-looseness is based on social norms. It is possible that cultural tightness reflects a sensitivity to norms in general, rather than just social norms. Similar to how some other broad aspect of culture like independence and interdependence associate with cognitive differences (analytical and holistic thinking, respectively; Varnum et al. 2010), tight societies may also be more sensitive to non-social norm violations (e.g., Proulx and Inzlicht 2012).

In the present paper, we show that Germans react to societal threats with stronger BIS-BAS activation compared to Russians. It leads to the question how Russians deal with threatening situations. In our studies, we showed that people from Russia reacted to threats with higher sadness than people from Germany. As mentioned above, Russians tend to pay greater attention to negative experiences (e.g., Grossmann et al. 2012). Such focus on negative experiences, including sad ones, seems to have positive implications for Russians and to help them to cope with problematic situations (Cote 1998). Furthermore, analysis of the open-ended questions in Study 2 revealed that Russians are more self-distant than Germans. It is possible that greater self-distance affords Russians to work through their negative experiences in an emotionally adaptive fashion, promoting insight and closure (Grossmann and Kross 2010). This speculation suggests an interesting avenue for future research on culture-specific way to cope with societal threats.

## Conclusion

Nowadays, Russian and German societies engage in numerous business and political interactions. To avoid possible misunderstandings during such interactions, one has to consider significant psychological differences between the two cultures, including culture-specific strategies of dealing with threat. The present paper shed some light on the ways people from Russia and Germany approach social issues. Culturally tight Germans showed higher BAS reaction to a threat that can be regulated by existing norms, and higher BIS reaction to a broader societal threat, as compared to culturally loose Russians. Russians, in turn, reacted to threatening situations with higher sadness. Our studies also highlight a conceptual and empirical difference between tightness and need for tightness, as well as the unique effects of the latter construct on regulation of societal threats. Altogether, the present research begins to demonstrate that tightness, need for tightness, and unique features of a threat-inducing stimulus play important roles for understanding how different cultures cope with societal threats. These findings can help us to find the most optimal way to deal with threatening situations and eventually promote efficient cross-cultural communication.

## Appendix 1

### The affect scale used in Study 1

To what extent do these words reflect your emotional state in the moment of described situation?

**Table:**

	Absolutely not				Absolutely
	1	2	3	4	5
Guilt					
Aggression					
Shame					
Forgiveness					
Need for punishment					
Leniency					
Sadness					
Admiration					
Anxiety					
Anger					
Proud					
Determination					
Confusion					
Nervousness					
Weakness					
Uncertainty					
Courage					
Satisfaction					

## Appendix 2

### The need for tightness scale used in Study 1 and 2

**Table:**

Totally disagree					Totally agree
1	2	3	4	5	6
1. I think there should be many social norms that people are supposed to abide by in [the country]					
2. I think people in [the country] should be punished, if they act in an inappropriate way					
3. I think people in [the country] should be agree upon what behaviors are appropriate versus inappropriate in most situations					
4. I think people in [the country] should always comply with social norms					

## Footnotes

1 Consistent with prior research (Gelfand et al. 2011), in our other work (Prokhorova et al. 2018), we have found evidence that Germany is significantly higher in tightness than Russia, *t*(127) = − 3.16, *p* = .002, *d* = 0.54 (*N*_GER_ = 66, *N*_RU_ = 63; *M*_GER_ = 4.22, *SD*_GER_ = 0.59, *M*_RU_ = 3.89, *SD*_RU_ = 0.62; answers were given on the six-point Likert scale). The study implied that Germany can be regarded as a tight and Russia as a loose culture.

2 The authors explore the serotonin transporter gene SLC6A4, in particular, a short allele of its promoter length polymorphic region known as 5-HTTLPR.

3 For exploratory purposes, we included two types of social transgressions to compare cultures in such reactions as shame and guilt, which are typical for self-caused wrongdoing but are not directly connected to BIS-BAS. Although the analysis confirmed that participants in the transgressor condition expressed higher shame and guilt than participants in the observer condition (*p*s < .001), we did not observe significant cultural differences in either shame or guild-related reactions (all *p*s > .406).

4 The whole sample consisted of 167 people from Russia and 173 people from Germany/Austria. However, some participants completed the survey following different instructions, which did not correspond to the aim of the present research. Specifically, they filled in the fourth part of the questionnaire (see description below) right after the first LBT. Thus, the delay between the first and the second/third LBT was too long to use the first LBT as a baseline.

5 In Study 1, we additionally administered the BIS/BAS-Trait Scale (Carver and White 1994), the International Personality Item Pool (Goldberg 2001), the Personal Need for Structure Scale (Thompson et al. 1992), and the Self-Monitoring Scale (Lennox and Wolfe 1984). Since BIS/BAS trait can be related to BIS/BAS state, we included it into analysis (*α*_RU_ = .75, *α*_GER/AU_ = .78). However, we did not find any significant main effects or interactions involving BIS/BAS trait, all *p*s > .10.

6 Despite our attempts to improve the internal reliability of the scale, it still stayed on a relatively low level. However, some literature justifies viewing the range of .5 to .6 as the minimally acceptable reliability level (see Peterson 1994).

7 In all analyses, we controlled for gender, age, and occupation. Inclusion of these covariates did not influence any of the reported results.

8 In Study 2, we additionally used the Transgression Scale (based on Gelfand et al. 2011), the RIC Scale (Kashima and Hardie 2000), the BIS/BAS-Trait Scale (Carver and White 1994), the Ethnocentrism Scale (Bizumic et al. 2009), five items about anti-Muslim dispositions, and eight items about an attitude to Russian policy. As in Study 1, we included BIS/BAS trait in preliminary analyses (*α*_RU_ = .78, *α*_GER_ = .67) but did not find any significant effects, all *p*s >.10. Since negative evaluation of transgressions might be a hallmark of cultural tightness, we also compared the two cultural samples with regard to the Transgression Scale (*α*_RU_ = .84, *α*_GER_ = .89). However, the difference between the cultures was non-significant, *t*(91) = 1.24, *p* =.22, *d* = 0.25.
